# The Utility of Faecal Biomarkers in the Discrimination Between Acute Gastroenteritis and Inflammatory Bowel Disease

**DOI:** 10.3390/biomedicines14091951

**Published:** 2026-08-30

**Authors:** Maria Ling Lundström, Christer Peterson, David Amcoff, Maria Lampinen, Henrik Hjortswang, Daniel Bergemalm, Charlotte Hedin, BIO-IBD-Consortium, Johan Dabrosin Söderholm, Lena Öhman, Jonas Halfvarson, Per Venge, Josef D. Järhult, Marie Carlson

**Affiliations:** 1 Gastroenterology Research Group, Department of Medical Sciences, Uppsala University, SE-751 85 Uppsala, Swedenmarie.carlson@akademiska.se (M.C.); 2 Clinical Chemistry, Department of Medical Sciences, Uppsala University, SE-751 85 Uppsala, Swedenper.venge@medsci.uu.se (P.V.); 3 Department of Pharmacy, Uppsala University, SE-751 23 Uppsala, Sweden; 4 Department of Health, Medicine, and Caring Sciences, Linköping University, SE-581 83 Linköping, Sweden; 5 Department of Gastroenterology, Faculty of Medicine and Health, Örebro University, SE-701 82 Örebro, Swedenjonas.halfvarsson@regionorebrolan.se (J.H.); 6 Department of Medicine, Karolinska Institute, SE-171 77 Stockholm, Sweden; charlotte.hedin@ki.se; 7 Department of Gastroenterology, Dermatovenereology and Rheumatology, Centre for Digestive Health, Karolinska University Hospital, SE-171 76 Stockholm, Sweden; 8 Department of Surgery, Linköping University, SE-581 83 Linköping, Sweden; 9 Department of Microbiology and Immunology, Institute of Biomedicine, University of Gothenburg, SE-405 30 Gothenburg, Sweden; 10 Department of Medical Sciences, Section of Infectious Diseases, Uppsala University, SE-751 85 Uppsala, Sweden; josef.jarhult@medsci.uu.se

**Keywords:** IBD, gastroenteritis, faecal biomarker

## Abstract

**Background**: Acute infectious gastroenteritis (GE) and inflammatory bowel disease (IBD) may have similar clinical characteristics at onset, and discrimination between these two diagnoses can initially be challenging. **Aim**: To explore faecal biomarkers reflecting neutrophil, eosinophil, and epithelial activity to distinguish between acute GE and IBD onset. **Methods**: Faecal samples were collected from patients presenting with acute GE (n = 28), treatment-naïve patients with newly diagnosed IBD (n = 40), and healthy controls, HC (n = 40). Faecal biomarkers, including faecal calprotectin (FC), myeloperoxidase (MPO), human neutrophil lipocalin (HNL), eosinophil cationic protein (ECP), and eosinophil-derived neurotoxin (EDN), were assessed by ELISA. The Kruskal–Wallis test was used for biomarker comparisons. To evaluate the discriminative capability of biomarkers, receiver operating characteristic (ROC) curves were established. **Result**: Patients with acute GE displayed elevated levels of all the tested biomarkers compared with HC (*p* < 0.001). Higher levels of faecal HNL (*p* < 0.001), EDN (*p* < 0.01), and MPO (*p* < 0.05) were seen in acute GE patients than in patients with newly diagnosed IBD. HNL yielded the highest discriminative capability between acute GE and IBD with AUC 0.74, 95%CI: 0.62–0.84. **Conclusions**: This exploratory study suggests that faecal biomarkers may provide complementary information in the differentiation of patients with suspected acute GE and new-onset IBD. Our findings indicate that faecal HNL may have the ability to distinguish between these conditions. However, given the exploratory design and limited sample size, findings should be interpreted with caution, and the discriminative value of HNL and the other biomarkers requires further validation in larger, well-characterised acute GE cohorts.

## 1. Introduction

Infectious gastroenteritis (GE) is commonly diagnosed based on characteristic clinical features, positive faecal bacterial culture, or polymerase chain reaction (PCR) assays detecting bacterial or viral DNA. However, distinguishing acute GE from new-onset inflammatory bowel disease (IBD) can be challenging at the time of initial clinical presentation.

A few studies, of which the majority are conducted in paediatric populations, have addressed the use of faecal biomarkers as diagnostic tools for GE. Most of these have focused on the biomarker’s ability to distinguish viral from bacterial gastrointestinal pathogens [1,2,3]. In contrast to patients with viral GE, patients with bacterial GE demonstrate increased levels of faecal calprotectin (FC) that can be distinguished from healthy controls (HC) [2,4]. However, these comparisons do not accurately reflect a diagnostic scenario where patients with acute onset of gastrointestinal symptoms, including diarrhoea, seek healthcare, and where both an infectious and inflammatory cause need to be considered. Since it is well-known that FC, the most established faecal biomarker for IBD, may also be increased in gastrointestinal infections, the outcome of FC analysis in these cases is often difficult for the clinician to interpret.

Calprotectin is a cytosolic protein originating mostly from activated neutrophil granulocytes in the gastrointestinal mucosa. FC is considered a sensitive but nonspecific marker for IBD, displaying elevated levels in many gastrointestinal-related inflammatory disorders, including acute GE [5]. This limitation of FC motivates the exploration of other biomarkers reflecting gastrointestinal inflammation. Myeloperoxidase (MPO) is secreted from activated neutrophils, but also from macrophages and monocytes in the intestinal mucosa. Human neutrophil lipocalin (HNL), also known as lipocalin 2 or neutrophil gelatinase-associated lipocalin (NGAL), is a protein of mostly neutrophil and macrophagic origin but is also released from epithelial cells [6]. Both MPO and HNL can serve as surrogate markers of intestinal inflammation when measured in faeces [6,7,8,9,10,11]. Eosinophil granulocytes are active in the intestinal mucosa in IBD, releasing toxic granule proteins such as eosinophil cationic protein (ECP) and eosinophil-derived neurotoxin (EDN) [12,13]. However, these proteins are also released in intestinal parasite infections where eosinophils participate in the host defence [14].

No published studies have examined the potential of FC or other faecal biomarkers to distinguish acute GE from new-onset IBD. Consequently, the value of these biomarkers in a clinical setting where infectious and inflammatory causes of acute gastrointestinal symptoms need to be differentiated remains unclear. This knowledge gap is of particular interest because these conditions often present similarly but require fundamentally different management strategies. Therefore, this study tested the hypothesis that FC, MPO, HNL, ECP, and EDN can differentiate between new-onset IBD and acute infectious GE.

## 2. Materials and Methods

### 2.1. Patient Cohort

Adult patients with clinical symptoms of acute GE were consecutively screened for study participation at their first healthcare visit for the ongoing illness episode at the Department of Infectious Diseases at Uppsala University Hospital, Uppsala, Sweden between April and November 2019. Exclusion criteria were previously diagnosed IBD (Crohn’s disease (CD), ulcerative colitis (UC), or microscopic colitis), coeliac disease, diverticulitis, colorectal cancer, or other non-infectious chronic disorders causing diarrhoea. Patients with gastrointestinal symptoms who were diagnosed with infections not typically considered causes of GE were also excluded. After obtaining informed consent, eligible patients were included, and faecal samples were collected. Demographic data were recorded, including a questionnaire covering frequency of diarrhoea and vomiting, and information on trips abroad in the last 3 months. To assess the aetiology of GE, faecal analyses were performed as per routine diagnostics, including bacterial culture (*Salmonella*, *Shigella*, *Campylobacter*, and *Yersinia enterocolitica*), PCRs (Norovirus, Enterohemorrhagic *Escherichia coli* (EHEC)), and a loop-mediated isothermal amplification (LAMP) assay targeting the C. difficile toxins A and B. In selected patients, for example, those with a history of travel to a risk country or drinking water from a private well, additional PCR analyses for intestinal parasites (*Cryptosporidium*, *Giardia intestinalis*, *Entamoeba histolytica*, and *Blastocystis hominis*) were conducted.

Treatment-naïve adult patients (aged ≥18 years) with endoscopically active, newly diagnosed CD or UC in the Swedish inception cohort in IBD (SIC-IBD) [15], who were consecutively recruited at the Department of Gastroenterology at Uppsala University Hospital, Uppsala, Sweden and Örebro University Hospital, Örebro, Sweden between November 2011 and October 2015, served as controls. At inclusion, demographic data were recorded, and blood and stool samples were collected. All patients underwent an endoscopic examination. The diagnosis of CD or UC was based on clinical, endoscopic, histological, and cross-sectional imaging according to internationally accepted guidelines [16,17].

A second control group of apparently healthy individuals was recruited via advertisements at the Department of Gastroenterology at Linköping University Hospital, Linköping, Sweden and Örebro University Hospital, Örebro, Sweden between October 2014 and September 2015 as a part of the SIC-IBD [15]. Controls were not allowed to have any history of current or chronic gastrointestinal symptoms and were all subjected to a flexible sigmoidoscopy with no macroscopic or histologic signs of pathology.

### 2.2. Measurement of Biomarkers and Microbiological Analyses

Collected faecal samples were transported on dry ice to Uppsala University Hospital, Uppsala, Sweden, and stored in a −70 °C freezer until analysis. Samples were thawed at room temperature and extracts were prepared in a single batch as described previously [8]. Faecal markers were measured using ELISA assays provided by Diagnostics Development, Uppsala, Sweden (www.diagnosticsdevelopment.com), assessing MPO (myeloperoxidase), HNL 763/764 (human neutrophil lipocalin), ECP (eosinophil cationic protein), and EDN (eosinophil-derived neurotoxin). All samples were measured in duplicate and after adjustment of water content in faeces, levels of markers were expressed as micrograms per gram of semi-dried faeces. The intra-and inter-assay variations were less than 12% for all assays. Correspondingly, FC was analysed with a chemiluminescent immunoassay using the LIAISON XL Analyzer (DiaSorin, Saluggia, Italy), Department of Clinical Chemistry, University Hospital, Uppsala, Sweden.

High-sensitivity (Hs)-CRP in serum was assayed at Uppsala BioLab, Uppsala Clinical Research (UCR) and Uppsala University Hospital, Uppsala, Sweden. Microbiological analyses were performed at the Department of Clinical Microbiology, Uppsala University Hospital, Uppsala, Sweden.

### 2.3. Statistical Analysis

Continuous variables were summarised using median and interquartile range (IQR). For the comparison of biomarkers between different groups, the Kruskal–Wallis test was used, followed by pairwise analysis of subgroups with post hoc analysis according to Conover. To test the biomarker’s discriminative capacity to separate acute GE from IBD, receiver operating characteristic (ROC) curves were constructed. Area under the curve (AUC) values were interpreted using commonly applied discrimination thresholds, with values of 0.7–0.8 considered acceptable, 0.8–0.9 excellent, and >0.9 outstanding [18]. Pairwise comparisons between AUCs were performed using DeLong’s test [19]. No adjustment for multiple testing was made. A *p*-value < 0.05 was considered significant. The MedCalc version 20.014 (MedCalc Software Ltd., Oostende, Belgium) was used for analyses.

### 2.4. Ethical Considerations

All patients and controls received verbal and written information and gave their written informed consent before participation. The study was approved by the Regional Ethics Review Board in Uppsala, Sweden Dnr 2011/065 (2019-01385) and Dnr 2010/313.

## 3. Results

### 3.1. Study Population

In total, 30 patients with suspected acute GE were enrolled and underwent microbiological testing. For 11 patients, no causative pathogen could be detected. After a careful examination of the patient’s record, 2 out of these 11 patients were excluded; one patient had symptoms lasting >4 weeks, classified as chronic, non-infectious diarrhoea, and the other patient had gastrointestinal symptoms associated with Dengue fever, not primarily considered to be a typical cause of GE. The remaining patients with negative cultures or PCRs (n = 9) all presented with typical self-limiting GE symptoms, probably reflecting other agents, not covered or missed by the standard testing. The results of microbiological testing for the 28 included patients with GE are shown in Table 1, and epidemiology as well as their reported symptoms prior to study inclusion are presented in Supplementary Table S1.

In the IBD group, 17 patients with Crohn’s disease (ileal n = 6, colonic n = 5, and ileocolonic n = 6) and 23 with ulcerative colitis (proctitis n = 8, left-sided colitis n = 9, and extensive colitis n = 6) were enrolled. In addition, 40 HCs were included. There was no difference in gender distribution between groups, but HCs were younger than patients with GE and IBD (*p* < 0.001), and CRP was higher in patients with GE compared to IBD (*p* < 0.05) and HC (*p* < 0.001). Baseline clinical characteristics for the study population are presented in Table 2.

### 3.2. Levels of Faecal Markers in Patients with Acute GE, IBD and HC

**Figure 1:**
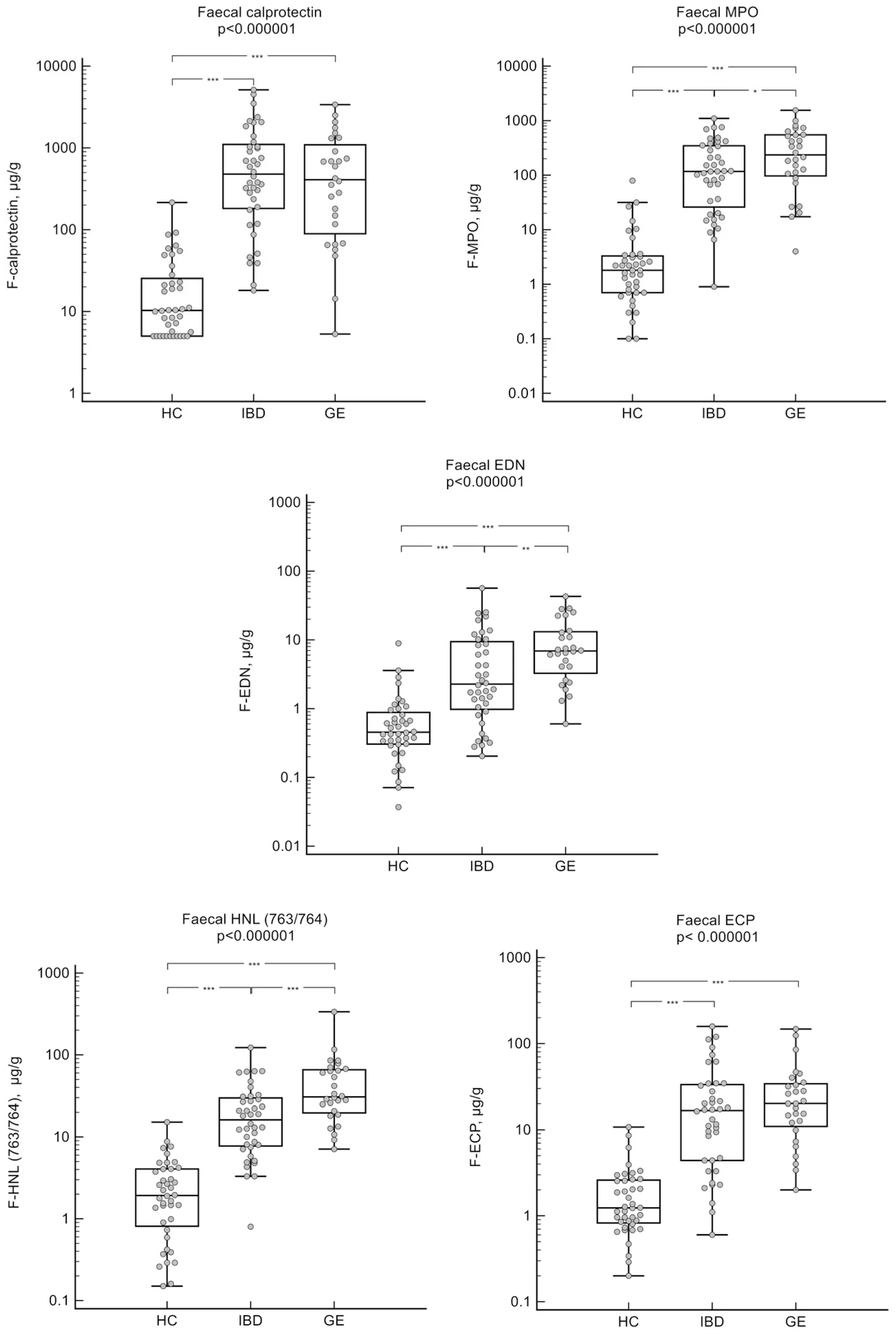
The box plots illustrate the median concentration, interquartile range (IQR), and T-shaped whiskers depict the minimum and maximum values (excluding outliers) of faecal biomarkers: calprotectin (FC), myeloperoxidase (MPO), human neutrophil lipocalin (HNL), eosinophil cationic protein (ECP), and eosinophil-derived neurotoxin (EDN) in healthy controls (HC), and patients with newly diagnosed inflammatory bowel disease (IBD) and acute gastroenteritis (GE). The Kruskal–Wallis test was used for group comparisons, followed by post hoc analysis (Conover). Significance is shown with bars (* *p* < 0.05, ** *p* < 0.01 and *** *p* < 0.001).

Biomarker levels for patients with GE, IBD, and HCs are shown in Figure 1. Patients with acute GE displayed higher levels of faecal HNL than patients with IBD (*p* < 0.001). Additionally, EDN and MPO levels were elevated in GE patients compared with patients with IBD (*p* < 0.01 and *p* < 0.05, respectively), whereas no differences were seen for FC and ECP.

Biomarker distributions across IBD subgroups (CD and UC) stratified by disease location and extent are presented in Supplementary Table S2. Due to the limited number of patients, subgroup analyses within the IBD cohort were not feasible.

Compared with HC, all biomarkers were significantly elevated in patients with acute GE (*p* < 0.001).

### 3.3. Faecal Markers for Differentiation of Acute GE from New Onset of IBD

**Figure 2:**
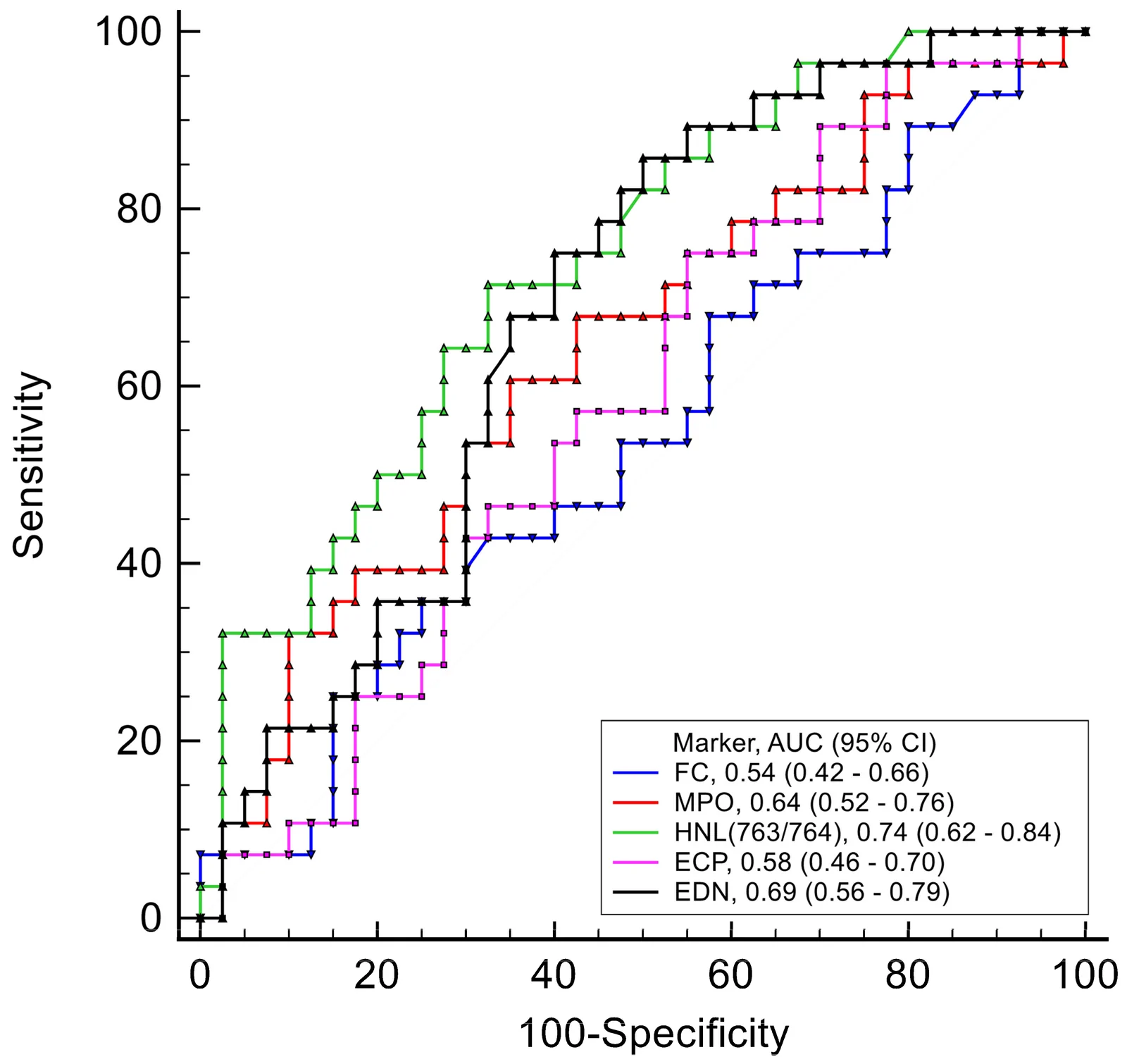
Receiver operating characteristic (ROC) curves for faecal biomarkers: calprotectin (FC), myeloperoxidase (MPO), human neutrophil lipocalin (HNL), eosinophil cationic protein (ECP), and eosinophil-derived neurotoxin (EDN) in patients with acute gastroenteritis (GE) versus patients with a newly diagnosed inflammatory bowel disease (IBD).

The biomarkers’ capacity to differentiate acute GE from IBD was analysed by the construction of ROC curves (Figure 2). HNL yielded the highest discriminatory capability with AUC 0.74, *p* < 0.001, followed by EDN (AUC 0.69, *p* < 0.004) and MPO (AUC 0.64, *p* < 0.04), whereas the corresponding analysis of FC and ECP did not demonstrate a significant discriminatory capability. Using DeLong’s test, HNL was better than ECP in distinguishing patients with acute GE from IBD (*p* = 0.014). No other biomarker showed superior discriminatory ability between the groups.

### 3.4. Biomarkers in GE Subgroups

**Figure 3:**
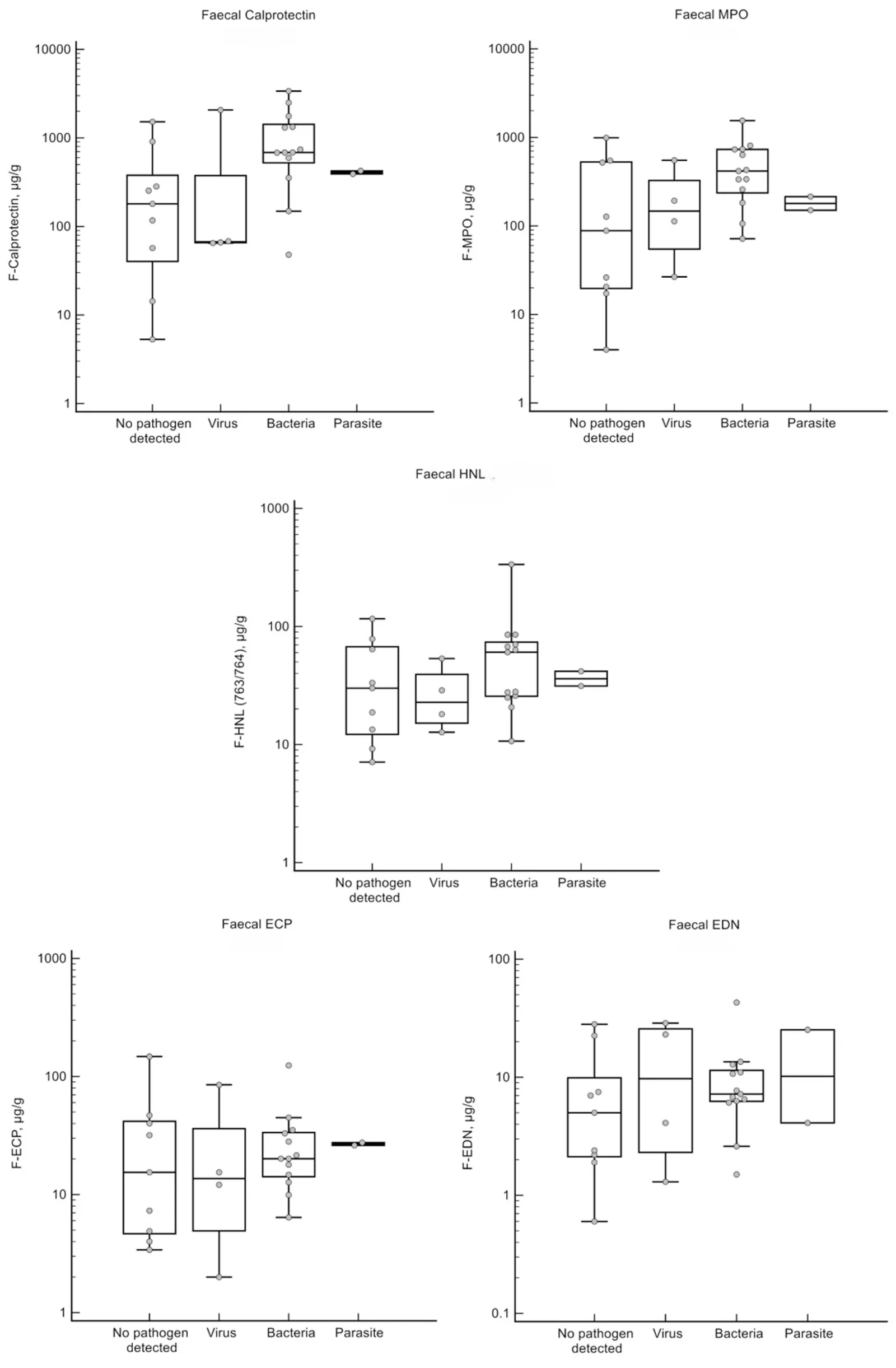
The median concentrations and interquartile range (IQR) of faecal calprotectin (FC), myeloperoxidase (MPO), human neutrophil lipocalin (HNL), eosinophil cationic protein (ECP), and eosinophil-derived neurotoxin (EDN) in patients with acute GE categorised by the identified causative pathogen (virus, bacteria, parasites, and patients with no pathogen detected).

Patients with GE were grouped according to causative GE pathogen: bacterial organisms (n = 13), viruses (n = 4), parasites (n = 2), and a group for patients with GE symptoms but with no pathogen detected (n = 9) (Table 1). Levels of faecal biomarkers in the different GE subgroups are presented in Figure 3. For comparison, biomarker levels in HC are shown in Supplementary Table S3. Due to the limited sample size of the subgroups, no statistical comparisons were performed.

## 4. Discussion

In this exploratory cross-sectional study, we analysed faecal biomarkers in a population of patients with acute GE and new-onset IBD and observed that faecal HNL appeared to have the greatest potential for discriminating these two conditions. Given the exploratory nature of the study, these findings should be considered preliminary and hypothesis-generating rather than definitive. Nonetheless, they raise intriguing questions regarding pathophysiology and potential differences in biomarker activity in infectious and immune-mediated gastrointestinal diseases. Activation of innate immune cells such as neutrophil and eosinophil granulocytes in the intestinal mucosa is central in inflammatory and infectious gastrointestinal conditions. Epithelial barrier disruption is a hallmark of many gastrointestinal infections, but barrier dysfunction, caused by microbial, environmental or genetic factors, is also a prerequisite for IBD development [20]. Considering the overlapping pathophysiological features between an acute infectious GE and the onset of IBD, increased levels of faecal biomarkers reflecting immune- and epithelial cell activity could be expected in both conditions (for GE, primarily those caused by bacteria).

The typical clinical course of infectious GE includes an acute onset of diarrhoea and/or vomiting that is usually self-limiting within a few days. In the vast majority of cases, the symptoms are so obvious that no further diagnostics are needed, and patients suffering from viral GE have often recovered before such analysis is complete. Nevertheless, there are clinical scenarios, e.g., patients with more longstanding gastrointestinal symptoms, where a new onset of IBD may be a possible differential diagnosis, and faecal biomarkers could play a role as diagnostic tools.

Studies examining faecal biomarkers in the context of gastrointestinal infections compared to IBD remain limited. Nonetheless, a recent study analysed FC in patients with an infectious GE and compared that with another group of patients with infectious GE, but who also had an established IBD diagnosis. While elevated FC levels were observed in the non-IBD group with infection, no further increase in FC concentrations was noted in the IBD population with concomitant infection [21]. As per this previous study, the clinical utility of testing FC in IBD patients with a suspected GE infection remains unclear.

Overall, in the current study, patients with acute GE showed high levels of faecal biomarkers, which, with the exception of FC, were higher than those observed in treatment-naïve, newly diagnosed patients with IBD. For faecal HNL, MPO, and EDN, the differences between GE and IBD patients were significant, with the most pronounced difference observed for HNL. Further, ROC curve analysis suggested that, although the magnitude of discrimination was modest, HNL may have greater discriminatory potential than the other biomarkers for distinguishing GE from IBD. In contrast, the corresponding analysis of FC did not discriminate between GE and IBD.

In the present study, we assessed HNL derived from neutrophils and epithelial cells, using an antibody combination directed towards specific HNL epitopes (763/764) [22]. Unlike FC or MPO, which primarily originate from neutrophils, HNL (763/764) therefore also reflects the impact of intestinal epithelial cells [6,23]. With this in mind, these findings suggest a potentially more dramatic effect on the epithelial cells of the infected patients than in patients with IBD. Based on the ROC analysis, it may be hypothesised that these two conditions in the acute stage may be separated using epithelial markers rather than markers mirroring neutrophil presence and activation (FC and MPO). In contrast to the current study, Thorsvik et al. did not demonstrate any difference in faecal NGAL (HNL) between patients with active CD or UC and patients with GE infections [23]. However, the authors did not clarify the NGAL antibody epitope-specificity, and the IBD patients included were not new-onset but chronically inflamed. One explanation for the discrepancy between studies could be that IBD in the acute setting may not involve epithelial cells to the same extent as IBD in a more chronic state.

Gastrointestinal infections exhibit diverse virulence mechanisms which involve the invasion of intestinal epithelial cells, cell disruption and initiation of an intestinal immune reaction [24]. Specific bacteria such as *Campylobacter jejuni* open cell–cell junctions in the epithelial layer, transmigrate through a paracellular route, and invade the epithelial cells. By intracellular signalling and release of toxins, they direct cells into programmed cell death and start an inflammatory cascade reaction through the participation of numerous immune cells [25]. On the other hand, Noroviruses are not believed to induce structural damage to the intestinal wall, but instead to affect secretory or absorptive functions, with only modest impacts on intestinal inflammatory activity [26]. Considering the varying virulence mechanisms of different GE microorganisms, different biomarker responses may be expected in subgroups of GE. However, due to the small sample size of our study, no reliable statistical analysis in the GE subgroups could be conducted. Only two patients were included in the group of parasite infections, both infected with *Cryptosporidium*. In this group, elevated levels of the eosinophil markers ECP and EDN would have been expected [27,28], and this was indeed observed in one of these patients. This individual displayed some of the highest recorded values of EDN among all GE patients analysed.

The approach of this study was exploratory, aiming to reflect a representative cross-section of patients with GE infections. Consequently, the limitations of the current study include a small sample size, which hindered subgroup analyses. Because of that, comparisons of biomarkers between patients with IBD and GE were performed without further division of GE patients based on microbial aetiology. To comprehensively assess the role of HNL and the other faecal markers in this distinction, larger patient cohorts enabling more robust statistical analyses are required. An additional limitation is that the analysed samples were derived from two different cohorts. Although identical protocols for sample collection, storage, and processing were applied to minimise handling-related variability, the samples had different storage durations. While long-term stability has not been formally investigated, our experience with these proteins has not provided evidence of significant degradation during prolonged storage. Therefore, any impact of differential storage time is likely to be limited, although it cannot be fully excluded. Furthermore, information on symptom duration prior to sample collection was not available. As samples were obtained at the patients’ first presentation to healthcare services, the interval between symptom onset and sampling may have varied between individuals and subgroups. This variability could have influenced biomarker levels and must be considered when interpreting the results.

The strengths of this study were the adult study population and the new-onset treatment-naïve IBD patients used for comparison, reflecting a real-world scenario.

In summary, we present the results of an exploratory study investigating a range of faecal biomarkers reflecting the activity of central cellular key players involved in intestinal infectious and inflammatory processes. Our findings suggest that faecal HNL may be of potential relevance in the discrimination of patients with acute GE and new-onset IBD. However, given the exploratory design and the limited sample size, the discriminative value of HNL in this context should be interpreted with caution and requires further validation in larger, well-characterised acute GE cohorts. Finally, although biomarkers may guide the clinician, clinical judgement is still essential for establishing a final diagnosis in these patients.

## Figures and Tables

**Table 1:** Microbiological diagnosis in patients (n = 28) with suspected acute gastroenteritis (GE).

Pathogen	n
Virus	
Norovirus	4
Bacteria	
Enterohemorrhagic *E. coli*	1
*Campylobacter* spp.	8
*Salmonella* spp.	1
*Shigella* spp.	1
*Clostridioides difficile*	2
Parasite	
*Cryptosporidium*	2
No pathogen detected	9

**Table 2:** Baseline clinical characteristics for individuals with acute gastroenteritis (GE), inflammatory bowel disease (IBD), and healthy controls (HC).

	Acute Gastroenteritis	Inflammatory Bowel Disease	Healthy Controls
n	28	40	40
Male/female	12/16	20/20	17/23
Age, y median (range)	44 (19–80)	41 (17–73)	26 (19–48)
CRP (mg/L), median (IQR)	22 (6.3–51)	4.9 (1.4–9.7)	0.6 (0.3–1.1)
CD		17	
L1/L2/L3/L4		6/5/6/1	
B1/B2/B3		16/1/0	
UC		23	
E1/E2/E3		8/9/6	

## Data Availability

The data set underlying this article cannot be shared directly under current legislation for data protection and must be requested directly from the respective holders after approval by the Swedish Ethical Review Authority.

## Supplementary Materials

The following supporting information can be downloaded at: https://www.mdpi.com/article/10.3390/biomedicines14091951/s1, Table S1. Summary of epidemiological characteristics and reported symptoms prior to study inclusion in patients with acute gastroenteritis (GE); Table S2. The median concentrations and interquartile range (IQR) of faecal calprotectin (FC), myeloperoxidase (MPO), human neutrophil lipocalin (HNL), eosinophil cationic protein (ECP) and eosinophil-derived neurotoxin (EDN) in IBD subgroups (CD and UC) with additional stratification by disease location and extent; Table S3. The median concentrations and interquartile range (IQR) of faecal calprotectin (FC), myeloperoxidase (MPO), human neutrophil lipocalin (HNL), eosinophil cationic protein (ECP) and eosinophil-derived neurotoxin (EDN) in healthy controls.

## Abbreviations

The following abbreviations are used in this manuscript:

IBD	Inflammatory bowel disease
GE	Gastroenteritis
HC	Healthy controls
FC	Faecal calprotectin
MPO	Myeloperoxidase
HNL	Human neutrophil lipocalin
ECP	Eosinophil cationic protein
EDN	Eosinophil-derived neurotoxin
