# Supplementary material for: The Utility of Faecal Biomarkers in the Discrimination Between Acute Gastroenteritis and Inflammatory Bowel Disease
Source: Biomedicines. 2026 Aug 30;14(9):1951. doi: 10.3390/biomedicines14091951 (PMC13604806; doi:10.3390/biomedicines14091951)
Supplement: Supplementary file 1 [file biomedicines-14-01951-s001.zip › biomedicines-4372598-supplementary.pdf]

Supplementary materials

Supplementary Table S1. Summary of epidemiological characteristics and reported symptoms prior to study inclusion in patients with acute gastroenteritis (GE).

	GE patients, n=28
Epidemiological characteristics	
Travelled abroad within the last 3 months, n (%)	18 (64)
Reported symptoms	
Vomiting, n (%)	5 (18)
Frequency of vomiting episodes/24h, Median (IQR)	6 (3–10)
Diarrhoea, n (%)	27 (96)
Frequency of diarrhoea/24h, Median (IQR)	10 (6–17.5)

Supplementary Table S2. The median concentrations and interquartile range (IQR) of faecal calprotectin (FC), myeloperoxidase (MPO), human neutrophil lipocalin (HNL), eosinophil cationic protein (ECP) and eosinophil-derived neurotoxin (EDN) in IBD subgroups (CD and UC) with additional stratification by disease location and extent.

IBD subgroup (n)	Faecal markers Median (IQR)				
	FC (μg/g)	MPO (μg/g)	HNL (μg/g)	ECP (μg/g)	EDN (μg/g)
CD (17)	589 (283-990)	118 (80.6-352)	13.1 (10.0-26.7)	17.1 (4.7-34.5)	1.9 (0.6-6.6)
CD Location					
L1 (6)	506 (236-834)	85 (47.5-109)	12.7 (10.6-18.9)	10.5 (5.7-12.8)	2.3 (0.6-5.7)
L2 (5)	695 (510-1390)	286 (119-352)	26.7 (13.1-27.2)	20.6 (20.1-34.5)	2.6 (1.9-13.7)
L3 (6)	479 (324-569)	114 (77.5-382)	10.1 (5.4-20.8)	10.9 (4.5-50.1)	2.2 (0.8-3.9)
UC (23)	359 (102-1105)	102 (17.9-274)	18.1 (6.6-31.8)	16.4 (2.8-30.1)	2.4 (1.3-9.5)
UC Extension					
E1 (8)	196 (39-711)	59.5 (14.1-152)	13.7 (5.6-22)	9.9 (2.3-21.2)	3.3 (1.5-7.1)
E2 (9)	359 (175-1180)	82.0 (33.5-420)	14.5 (7.5-40.2)	18.0 (10.6-27.8)	8.4 (1.4-12.0)
E3 (6)	702 (337-1810)	163 (44.5-307)	20.1 (10.6-29.9)	15.5 (4.2-31.1)	1.6 (1.3–2.1)

Supplementary Table S3. The median concentrations and interquartile range (IQR) of faecal calprotectin (FC), myeloperoxidase (MPO), human neutrophil lipocalin (HNL), eosinophil cationic protein (ECP) and eosinophil-derived neurotoxin (EDN) in healthy controls.

Faecal markers<br>Median (IQR)	Healthy controls<br>n=40
FC (μg/g)	10.3 (5.0-25.4)
MPO (μg/g)	1.80 (0.70-3.30)
HNL (μg/g)	1.92 (0.81-4.06)
ECP (μg/g)	1.24 (0.82-2.60)
EDN (μg/g)	0.45 (0.31-0.88)
